# Expanding the pragmatic lens in implementation science: why stakeholder perspectives matter

**DOI:** 10.1186/s43058-025-00730-z

**Published:** 2025-04-23

**Authors:** Richard Boulton, Antonina Semkina, Fiona Jones, Nick Sevdalis

**Affiliations:** 1 School of Health & Medical Sciences, City St George’s, University of London, London, UK; 2 Faculty of Health, Science, Social Care and Education, Kingston University, London, UK; 3 NIHR Policy Research Unit in Health and Social Care Workforce, The Policy Institute, King’s College London, London, UK; 4 School of Health & Medical Sciences, City St George’s, University of London, London, UK; 5 Centre for Behavioural and Implementation Science Interventions, National University of Singapore, Singapore, Singapore

**Keywords:** Pragmatism, Implementation science, Psychometrics, Pragmatic measures, Stakeholder engagement, Implementation outcomes, Implementation determinants, Patient and public involvement

## Abstract

**Background:**

Pragmatism is important in implementation science to ensure that implementation methods reflect the practical concerns of the stakeholders and services involved in change. To evaluate the usability of these methods, pragmatic measures have been developed using psychometrics. However, existing approaches have predominantly inherited a definition of pragmatism from the evidence-based healthcare movement. These metrics may not reflect concerns with pragmatism that public stakeholders (defined as those with expertise by experience of healthcare systems) may have with implementation science.

**Aims:**

Consequently, our aim was to carry out participatory research to explore stakeholder views of pragmatic measures in implementation science theory.

**Methods:**

We convened a working group of eight stakeholders. To facilitate discussion, we created educational materials, including a video and flyer. The working group conducted three meetings, engaging in abductive analysis to investigate the presented issues.

**Results:**

Stakeholders expressed concerns about the restricted definition of pragmatism, the potential for biases in measurement, and the necessity for a holistic, pluralistic approach that incorporates diverse perspectives when developing and evaluating implementation theory and metrics. These findings underscore the risk of distorting the development of implementation science methods without the input and scrutiny of stakeholders. Neglecting the wider application of pragmatic philosophy in implementation science could limit stakeholder involvement in the design of implementation methods and service transformation.

**Conclusions:**

This study, guided by experts with lived experience in healthcare services, opens doors for considering pragmatic philosophy in the evolution of pragmatic implementation measures and metrics, offering numerous promising directions for further exploration.

**Supplementary Information:**

The online version contains supplementary material available at 10.1186/s43058-025-00730-z.

Contributions to the literature

- Pragmatism in implementation science is defined by evidence-based methodologies.
- Current definitions of pragmatism in implementation science are not reflective of wider definitions of pragmatism in the social sciences.
- Stakeholders emphasise that pragmatism could be considered from wider perspectives to enhance inclusion in implementation methods.
- Incorporating pragmatic philosophy in implementation science would enhance methodological accuracy, interdisciplinary compatibility, and stakeholder involvement.

## Background

Recent developments in implementation science have underscored the significance of pragmatism, emphasising the need for research to align with ‘real-world’ practicalities [1]. This trend reflects a broader shift in evidence-based healthcare towards practical, usable measures rooted in practice [2].

Implementation science has primarily focused on two areas: firstly, the development of embedding methods or frameworks in practice, such as pragmatic trials or RE-AIM [3, 4].^1^ And secondly, the creation of pragmatic implementation measures which aim to evaluate the pragmatic qualities of implementation measures, exemplified by tools like ‘the Psychometric and Pragmatic Evidence Rating Scale’ (PAPERS) [5]. Where the former is a straight forwards task of relating programs or treatments to practice, the latter task of evaluating pragmatism poses more of a challenge, because firstly, there is an added level of abstraction in measuring the measures that are used in practice. And secondly, in the words of Glasgow et al.: “there is no [accepted] way of universally evaluating pragmatism” [2].

Our team conducted a recent scoping review on defining pragmatism in implementation science, which revealed a lack of coherence in the field's use of the term [6]. Only nine papers discussed pragmatism, with limited stakeholder involvement in developing pragmatic measures like the PAPERS rating scale. Typically, assessments of pragmatism relied on expert panels and psychometrics [7–9],^2^ neglecting input from diverse stakeholders, including patients and service users, resulting in a narrow focus and potential oversight in methodology.

Pierce’s original maxim of pragmatism states:"Consider the practical effects of the objects of your conception. Then, your conception of those effects is the whole meaning of the conception"[10]. For this study, it can be taken to mean that the challenge of evaluating pragmatism lies in the constantly changing social dynamic between real-world scenarios and pragmatic measures. The creation of measures may make ideal principles detached from practical realities and stakeholder concerns [11]. Any use of a scale is an attempt to place a theory on to a more complex reality [12]. Psychometric scales therefore, cannot avoid remaining open to the possibility of their measurement being inaccurate [13]. Additionally, methodological biases may favour certain measurement methods and forms of expertise, neglecting diverse perspectives and exceptional cases [14].

Addressing these issues requires expanding conceptions of pragmatism and incorporating diverse voices and perspectives into the measurement process. Broader discussions may explore the relevance and inclusivity of implementation science methodologies and prompt considerations on balancing reflexivity, fidelity, and adaptivity [15–17].

Therefore, questioning the conceptualisation of pragmatism directly engages the flexibility and inclusivity of implementation measurement design, and the extent to which they are guided by both professional expertise and lived experience. Considerations of pragmatism may demonstrate why channels of participation, reflection, and interpretation are an important accompaniment to any evaluation of a measurement’s pragmatic qualities.

## Aims

This study aims to explore definitions of pragmatism in implementation science with public stakeholders, focusing on how pragmatism is evaluated by pragmatic measures. Specific objectives include:

- Exploring how to measure pragmatism from the point of view of stakeholders.
- The theoretical implications of bringing in a wider understanding of pragmatism for pragmatic implementation research.

## Methods

We formed a diverse working group comprising stakeholders (defined as those with expertise by experience of healthcare systems)^3^ to engage in discussions regarding pragmatism within implementation science. Our approach aimed to frame the problem effectively and foster meaningful debate among stakeholders, ensuring their active involvement in the research process.

### Framing the problem

Initially, our strategy involved attempting to validate the pragmatic constructs of existing psychometric scales in the field by seeking input from patients and the public.^4^ However, feedback from a Patient and Public Involvement and Engagement panel revealed potential limitations with this approach.^5^ It was noted that such an approach could inadvertently steer discussions towards merely validating existing measures rather than engaging participants in a deeper exploration of the research process itself [18–20]. This insight prompted us to develop user-friendly informational resources to address these concerns and provide a foundation for informed discussions among participants.

To ensure accessibility and comprehensibility, we designed a set of non-technical informational resources, including a short AV presentation, a concise flyer, and a worksheet of consideration points (appendix 1–3) [21–23]. These materials aimed to introduce key concepts such as implementation science, pragmatism, pragmatic measures, and PAPERS in a clear and understandable manner, while also highlighting the importance of diversity and wider representation in research endeavours. Our multimodal approach aimed to cater to diverse learning styles and personal perspectives, thereby facilitating more inclusive and engaging discussions within the working group [24–26].

### Working group debate

The informational resources were shared with a public research panel for feedback and further revisions to enhance inclusivity. Subsequently, we employed a targeted recruitment strategy to assemble a diverse group of participants. This involved advertising our project through various public research networks, including King’s Improvement Science, the National Institute for Health Research (NIHR) Applied Research Collaboration (ARC) South London, and Shaping Our Lives.^6^ To attract individuals with a range of perspectives and firsthand experiences of healthcare systems [27, 28]. And include viewpoints that may not have had the chance to directly reflect on pragmatic measures before. Potential participants were invited to complete a selection form to ensure as much demographic and experiential diversity as possible within the working group (Table 1, appendix 4) [29].

**Table 1:** Demographics of the PPI group members

Gender	62.5% female, 37.5%male
Ethnicity	50% White, 37.5%Black, 12.5% Mixed
Age	25% − 21–30 y.o., 25% − 51–60 y.o., 25% − 61–70 y.o., 12.5% − 31–40, and 12.5% − 80 +
Marital status	75% single, 25% in civil partnership
Education	12.5% had A levels, 25% had vocational education diplomas, 37.5% had (or studied towards) BA, 12.5% had a MA and 12.5 had a PhD
Disability	75% had a disability
Service use experience	75% were current or previous service-users, 62.5% were current or previous carers, and 25% were close to a service-user or a carer

Meetings were conducted using Microsoft Teams to maximise accessibility and accommodate the diverse schedules of participants. We organised three one-hour discussions over three weeks, limiting the group size to eight members to facilitate in-depth exchanges and meaningful contributions from all participants [30, 31]. Members received compensation for their time to ensure equitable participation and acknowledge the value of their input [32].

Throughout the working group meetings, the research team created a supportive and inclusive environment conducive to open dialogue and meaningful engagement [33]. Discussions were structured (around themes taken from PAPERS) to encourage participants to delve deeply into the subject matter, interact with each other's perspectives, and build upon shared insights over time (appendix 2). Topic guides were provided in advance to facilitate focused discussions around key themes and questions related to the concept of pragmatism in implementation science [34].

### Analysis

Debates were recorded, transcribed, and analysed in NVivo using abductive analysis [35, 36]. Codes were created using abductive reasoning by RB in 3 stages. (1) iterative movement between a close reading of the data and theoretical concepts from pragmatic philosophy to create a code book. (2) Abductive data reduction through coding equations to refine and structure the codes. (3) In-depth abductive qualitative analysis to explore relationships between coded data and pragmatic philosophy. At each stage the coding book was shared, discussed, and verified in research team meetings. This was to ensure the accuracy, understandability, and relevance of the themes as they emerged (appendix 5). The final paper was shared with participants, and they were asked if they would like to be included as co-authors. The GRIPP2 checklist was used to ensure the quality of the report (appendix 6) [37]. The findings section below summarises the themes agreed.

## Findings

Stakeholder discussions highlighted 6 themes. The participant quotes that informed the themes are compiled in Table 2.

**Table 2:** Coding table

Theme 1	
Complexity of the Subject Matter	“These proposals are very challenging […] my little grey cells are throbbing.” (Rory, email correspondence in preparation for discussion 2)
Complexity of the Subject Matter	“It means there’s work to be done if in actual fact the people you've got on your panel are saying: Actually, I don't understand it [refereeing to PAPERS].” (Quin, discussion 2)
Complexity of the Subject Matter	“It's because it's [referring to pragmatism + psychometrics] hard to interpret, mostly because it's the wording and everything seems a little more complex.” (Frankie, discussion 2)
Complexity of the Subject Matter	“an outcome is an easier concept for most people to understand [than a measure] because that's what we're, you know, we hope to achieve when we engage with anything we hope to see an outcome at the end of the day. So I think that would be easier for, say myself to respond to, and it's also what most people are used to.“ (Bobbie, discussion 2)
Complexity of the Subject Matter	“[outcomes are] really important, we would hate. I'd hate to think that you know, we were doing work and we weren't looking at the outcomes of work.” (Quin, discussion 2)
Complexity of the Subject Matter	“Simplifying terms [of measurement methodology] makes it more attractive to contribute, and it also helps. You have some concepts [that] are overwhelming and phrased in a complex way. If you find a way to restructure it. It really helps us to understand and contribute to the discussion.” (Frankie, discussion 3)
Complexity of the Subject Matter	“Yeah, just placing things in the practical is really important. I've seen some researchers doing very good papers, but no one understands them. And so having a really good and practical aspect is so important.” (Charlie, discussion 3)
Theme 2	
Weighting pragmatism	“I was just thinking, you know, you review the evidence base of what's been done before and what has and hasn't worked, and you build on that by trial and error. But I know that's a pretty simplistic answer, but it's a general answer” (Sam, discussion 3)
Weighting pragmatism	“I really think that what Rory’s example highlights is the need for, with our measures of any kind, of having the right blend of mathematical and qualitative and indeed relational components to the assessment when measuring outcomes.” (Sam, discussion 2)
Weighting pragmatism	“I was just thinking it's like a continuum and it very much is the subjectivity continuum. I don't want extremes. You get something which is wholly subjective and potentially unreliable and capricious. So the other end you've got at the very end, you've got objectivity and most of the time we're somewhere in between. But you can test and validate things to see which end their nearest if you like.” (Sam, discussion 3)
Weighting pragmatism	“For me, it's like setting a condition that ‘if’—about the usefulness. But it doesn't limit to this condition and makes me wonder what other cases could be except this one. What are the conditions about the usefulness. ‘Should be only useful if’—it is not a statement like that. It leaves an open window and that makes it somewhat incomplete as a statement because it lists only one condition. What are the others? And that is up for discussion.” (Adrian, discussion 1)
Weighting pragmatism	“Co-production is so important to get the perspectives of everyone and then you don't sit in your ivory tower, produce things that are not relevant for anyone.” (Charlie, discussion 3)
Weighting pragmatism	“I was just going to add. [co-production] also allows for a particular thing to be designed fit for purpose. It shapes things to be. More relevant.” (Bobbie, discussion 3)
Theme 3	
Bias	“in predictive terms, it's [referring to psychometrics] very, very dependent on the nature of the data it's trained on and the nature of the mechanisms whatever they are, clinical or otherwise, that actually leave, produce that kind of data. Because if you've got a very dynamic mechanisms which evolve over time because they're dependent on so many external factors, then any given [scale] will only have a very limited shelf life.” (Sam, discussion 1)
Bias	“I don't have the skills to speak Bengali. I don't have the skills actually that have the connections with the mosques and to be able to have that really great communication. So I couldn’t hold events there. I didn't have the skills, that those volunteers had on that project. So you know me running the project would have really coloured the project in a certain way whereas in actual fact we got a load of other stuff back because in actual fact it was a whole range of people that lived in those communities that helped to run that project.” (Quin, discussion 2)
Theme 4	
Holism	“in the context of what's called phase four clinical trials is so important to measuring outcomes. But also, I think and I don't want to complicate things, but I'm aware of this complexity. The fact that we're not just biological clinical individuals, we're social, relational individuals. And so it's, you know, remembering that outcomes can, you know, not just be at the symptom level but at the relational and quality of life and work and other levels. And also they extend beyond the individual you know to their family and friends and fellow workers and everyone else you know, with whom they have meaningful interactions. Just an extra layer of complexity which I apologize for.” (Sam, discussion 1)
Holism	“I think when, we involve humans in a qualitative [study], there's an engagement, which means that we're doing something else than just creating a formula which we think is quite good on some levels. And I think that there's something about what we're doing here. You're involving patients. We're being involved with you in the implementation of science. So when we actually ask people about certain things, maybe about their health or their inequalities or about how they would like services to be shaped.” (Quin, discussion 2)
Theme 5	
Plurality	“I'm looking at it as perspective that the results from implementation outcomes could be used to create a model. You understand, these implementation [scales] could actually look more into specific individualized cases.” (Morgan, discussion 1)
Plurality	“I ran a project in [the council] around [children] and raising awareness around cancer, and early diagnosis and with public health […]. So they wanted these amounts of numbers, this amount from this group, and this amount from that group and stuff like that. But we were saying, well, we were getting lots of anecdotal information from patients and we're not collecting it, we're not using it. And in a way, we're not valuing it. What people say because naturally a lot of time, like us, with this information, it's quite hard to understand. But you know, people do understand a lot about their own health. People do understand. Sometimes what they need, and so sometimes they just need to be asked for that And I just feel that, I mean it's a difficult one because, you know [its] kind of subjective. But I don't know that's the problem for me that you know it is subjective. I think it's good to speak to different groups of people have a different take on something, and sometimes that can affect us all as well. So I just think it fleshes out..” (Quin, discussion 2)
Theme 6	
Perspectivism	“Perspective, which may be culturally different. Uh, and therefore you have to ensure that what may be a minority or majority prefers and I suppose there are perspectives within clinical practice that may be comparable to. And the example which springs to mind that somebody's freedom fighter may also be another person's terrorist, and I suppose within clinical care. It may be your belief, for instance. From a Jehovah's Witness point of view that they do not want an intervention under any circumstances because that is what they believe.” (Rory, discussion 2)
Perspectivism	“There are obviously inevitable problems with utilitarian ethical perspective, because at the end of the day, you may not end up with the greater good because it could be that most people aren't affected by that certain thing, but very few may be affected very highly. So it's, you know, before you apply utilitarianism, you have to consider what's on the ground and who might be affected by what and how and to what extent.” (Sam, discussion 2)

### Complexity of the subject matter

Participants universally acknowledged the complexity and intellectual challenge inherent in the subject matter of psychometrics, pragmatic measures, and pragmatic philosophy. Participants had different levels of familiarity with the methods and techniques introduced. Difficulty in understanding the PAPERS scale, particularly its abstraction, was a common sentiment. Participants expressed a preference for discussing tangible outcomes rather than abstract measures and constructs.

### Weighting pragmatism

While recognising the importance of psychometric pragmatic measurement constructs, participants hesitated to judge the relative importance or propose alternatives to constructs in the PAPERS scale. The feasibility of ranking or rating outcome measures was questioned due to potential bias, limitations, and subjectivity. Discussions highlighted the need for a balanced approach, incorporating qualitative components when quantitative measures and scales are used.

### Bias

Participants raised concerns about potential bias that may creep into fixed scales or measures over time. They emphasised how pragmatism requires dynamic thinking to remain representative and the need to maintain interpretation in measures to mitigate bias. Some participants shared experiences from projects in diverse communities which underscored the importance of inclusivity and acknowledging different viewpoints.

### Holism

Participants highlighted the holistic nature of human experiences, emphasising that the multidimensional aspects of being human are not fully reduced to clinical symptoms and measurement scales. Discussions highlighted the need to consider social, relational, and quality of life factors in measure design. Participants acknowledged the limitations of formulating a pragmatic scale that accurately captures human complexity.

### Plurality

Participants stressed the importance of incorporating diverse perspectives in to evaluating measures, emphasising the value of considering pragmatism on an individual case by case bases. A participant shared a further example from a past project that highlighted the pitfalls of overlooking diverse perspectives, where one specific person’s perspective in one moment may speak more universally for us all at larger scales and over a longer span of time.

### Perspectivism

Discussions reflected on the complexities of reconciling differing perspectives, particularly in culturally diverse contexts. Participants cautioned against adopting a one-size-fits-all approach to pragmatism, recognising the inherent subjectivity in value judgments. Some considered measurement scales to be utilitarian in their approach to ethics, that may result in decision-making processes where the perspectives of the many are considered over the few.

Combined, the findings underscored the need for a nuanced and multifaceted approach to measuring pragmatism in implementation science. The need for inclusive, participatory, and adaptable approaches to measurement emerged as a key theme, reflecting the complex interplay of dynamic factors influencing what it means to be pragmatic.

## Discussion

The themes arising from the discussions revealed public perspectives on both pragmatism and how to measure pragmatism in implementation science. Despite their varying levels of familiarity with expert debates in evidence-based methodologies or implementation science, participants raised pertinent issues that highlight the partiality inherent in the concept of pragmatism employed in Implementation Science.

The themes echo broader discussions in scholarly literature, particularly within American Pragmatism, which has informed the development of research methodologies distinguishing between qualitative and quantitative approaches [38]. While some methodological strands, such as mixed-methods, middle-ranged theory, and science and technology studies, have incorporated pragmatic philosophy, implementation science methodologies have yet to consistently integrate these perspectives [39].

### Inconsistencies in pragmatism

While some sources (such as 1) acknowledge the importance of stakeholder involvement in defining pragmatic measures, discussions of pragmatism remain confined to quantitative methodologies. Implementation science's commitment to evidence-based methods, prioritising objective realities over subjective ones, further exacerbates these inconsistencies [40].

The reliance on evidence-based methods to validate pragmatism poses challenges. Evidence-based methods prioritise the ‘evidence’ from certain voices and interpretations over others, potentially leading to discrimination [41]. Pragmatic measures prioritise actionable research evidence rather than a fully accurate exploration of reality, creating a potential for mismatch between the idealised pragmatism of psychometric constructs and pragmatic realities as encountered during implementation, and coming to bear on diverse stakeholders [42].

This raises questions about whether implementation science methodologies should exclusively adhere to evidence-based principles. Public stakeholder concerns may be difficult to assimilate into evidence-based hierarchies without losing effectiveness, yet they offer valuable insights into practical implementation challenges and priorities [43]. Embracing pragmatism may necessitate a revaluation of evidence-based methods in favour of more practical approaches, such as participatory action research or co-production [44, 45].

### Scales and pragmatism

While the use of scales like PAPERS is not invalidated and remains a useful tool, relying solely on psychometrics may overlook subjective considerations crucial to the implementation process [46, 47].^7^ Other qualitative approaches should be considered where possible when evaluating implementation measures. Stakeholder perspectives underscore the importance of including diverse voices in discussions on measurement methodologies, highlighting issues that should be addressed in formative discussions on methodology shaping.

The full implications of pragmatism cannot be encapsulated in a formula or rating scale. Decision-making must continuously evaluate diverse concerns and perspectives within specific situations. Involving public stakeholders in measurement evaluation and method design requires ongoing, co-produced techniques that acknowledge the multifaceted nature of pragmatic decision-making [48].

### Wider implications for implementation

Although this study has focused on pragmatic measures, there is a wider implication to the discussion and application of the methods. Stakeholders should be better considered not only in the implementation of interventions, but in the formulation of our methods, measures, strategies, and as partners in research more broadly.

The design of many implementation science resources limits exploration of pragmatism, often assuming that wider interpretation is problematic to arriving at concise research evidence. There is a need for a broader examination of research methodologies and their representation in the field to better integrate implementation science aims with partners'needs [49].

As implementation science frameworks continue to expand, there is a growing need to emphasise openness to interpretation, accommodation of conflicting perspectives, and promotion of reflective practice [50]. This study contributes to discussions on the representativeness and usability of implementation science theory by highlighting the misalignment between wider stakeholder perspectives and the current use of pragmatism in the field.

### Strengths and limitations

Engaging stakeholders on complex topics like pragmatic measures and evidence-based methodologies is a significant challenge, and simple questionnaires risk tokenistic engagement. We chose to educate and involve fewer participants more deeply rather than assemble a larger, less engaged group. The diverse 8-member panel aimed to represent marginalised viewpoints but cannot encompass all perspectives possible. While the article format reflects group discussions, it provides only a glimpse into public concerns about pragmatic measures. Future research could use the identified themes to explore nuances in implementation science methods/theory and pragmatism.

## Conclusion

Different approaches and different methods may account for different definitions of pragmatism. There is no one method which may account entirely for a given reality in every circumstance. The pragmatic, practical measures sought after to validate evidence-based methods may not correspond to the pragmatic ‘real world’ observed when employing methods to increase participation and inclusion in the implementation process. As practice and the practical are a key component in any instance of implementation, implementation science should conduct a more thorough and inclusive appraisal of what pragmatism means in the process of creating measures and scales, i.e. if psychometric measures are used to evaluate scales how and where do further participant voices come into the future application of scales.

When contemplating more broadly what pragmatism means, some things to consider are how implementation methods can be made more direct and use inclusive language and procedures. Any methods used should attempt to engage public stakeholders in reflections from the ground up and not just from the top down. Methods should minimise obfuscatory language or research processes that divert localised discussion, dialogue, and decision making in measuring outcomes. Where complex clinical measures are warranted, they should be combined with other methods to ensure meaningful engagement with ‘real world’ considerations not biased to validating any one methodological representation.

Wider pluralistic exploration of pragmatism in implementation method design may uncover service realities not reducible to (and obscured by) a focus on evidence.

## Supplementary Information

Supplementary Material 1.

Supplementary Material 2.

Supplementary Material 3.

Supplementary Material 4.

Supplementary Material 5.

Supplementary Material 6.

## Data Availability

The datasets generated and/or analysed during the current study are not publicly available due to participant confidentiality but are available from the corresponding author on reasonable request.

## Footnotes

1 also see PRECIS.

2 PAPERS takes a quantitative approach using psychometrics (a technique of mathematical modelling) to give implementation measures a rating (or score) on a Likert scale on their pragmatic qualities.

3 For clarity and consistency with existing literature, the term ‘stakeholder’ is used in this paper to refer to efforts to include patient and public perspectives in the research process, ensuring diverse knowledge and expertise. Whilst acknowledging that an ideal group of who exactly is a ‘stakeholder’ cannot be definitively defined.

4 We initially questioned a pragmatic way to rate scales in the Implementation Outcome Repository https://implementationoutcomerepository.org/ or COSMIN https://www.cosmin.nl/ .

5 See ARC South London Public Research Panel: https://arc-sl.nihr.ac.uk/about-us/nihr-arc-south-london-public-research-panel

6 See King’s Improvement Science https://kingsimprovementscience.org/involving-the-public ARC South London https://arc-sl.nihr.ac.uk/involving-patients-public and Shaping Our Lives https://shapingourlives.org.uk/about/

7 E.g. additional ways of rating scales in the Implementation Outcome Repository may involve facilitating collaborative fora and the qualitative appraisals of scales.
