# Supplementary material for: Expanding the pragmatic lens in implementation science: why stakeholder perspectives matter
Source: Implement Sci Commun. 2025 Apr 23;6:48. doi: 10.1186/s43058-025-00730-z (PMC12016074; doi:10.1186/s43058-025-00730-z)
Supplement: Supplementary file 1 — Supplementary Material 1. [file 43058_2025_730_MOESM1_ESM.docx]

Evaluating measures of Implementation outcomes

# Background

**Implementation science seeks to take evidenced interventions and implement them into practice.** For example, an evidenced intervention may be that screening high risk patients for lung cancer reduces lung cancer mortality. Implementation science is the strategies used to put that evidence into practice. Services wishing to implement this evidence into practice would create an implementation strategy to do so.

**To see if implementation has been achieved and how well it has been achieved the outcome of an intervention needs to be measured.** There are many outcome measures available depending on what needs to be known about the implementation strategy. For example, in lung cancer screening, one of the most important outcomes we may wish to look at is if the strategy has indeed lowered mortality, but there may also be other outcomes of interest including: how thoroughly the service implemented the evidence, if implementation was arduous or not, whether it caused any unforeseen consequences, or if the screening was sustained in the long term.

**There is a great need therefore, to ensure that the outcome measures used are practical, easy to use and applicable to the real world (i.e. pragmatic)** to ensure that implementation strategies are well guided and can demonstrate their achievements effectively.

To do this, **Implementation science has been attempting to come up with ways to assess how usable outcome measures are** by creating another measure. One that can be used to measure the quality of outcome measures (confusing we know!). These have been called pragmatic measures. **Pragmatic measures are designed with the goal of evaluating the usability of outcome measures.** However, the development of pragmatic measures is in their early stages and leave open many questions about how best to go about assessing if an outcome measure is practical.

**The current development of pragmatic measures is to create a psychometric scale;** a tool that attempts to rate pragmatic aspects of an outcome measure on a numerical scale. This is not as straightforward as it would seem however, because when implementing evidence into practice we are making interventions into complex services that have complicated systems of values that determine whether any changes to a service are an improvement overall or not. **Many values are subjective and involve the interpretation of implementors, practitioners or service users to determine which values should be prioritised over others.**

# Summary of the problem

Evidenced interventions need to be **implemented** into practice


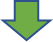


**Implementation outcomes** need to be **measured**


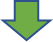


The measurements need to be **usable**


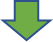


The **usability** of measures needs to be **evaluated**


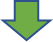


Currently usability is evaluated **quantitatively** (e.g., rating scales)


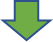


Usability also needs to be evaluated **qualitatively** (e.g., open-ended questions) to capture more context and nuances

# Search for solution

| **Numerical scales (psychometrics) do not produce fully objectives results, because they are designed to summarise situations with many complicated variables into one answer.** For this reason, the numerical scales that we use are sometimes criticised for being overly simplistic and assuming that it can make measurements without considering wider contexts of people’s lives, values, and ethics. As Implementation science has used no other method than psychometrics to measure usability and pragmatism the resulting perspective is not very open. There has been little attempt to diversify the expertise that is evaluated when compiling measures that are pragmatic. Such as patient and service user expertise.  **We propose to start a discussion with you around the assumptions used by implementation science to define pragmatism. The results will be open ended and used to spark discussion on how to measure implementation outcomes.** | *Here’s an example of how a numerical scale generally looks like:*  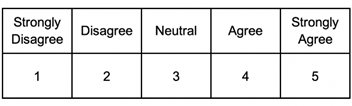 |
| --- | --- |

# Aims

*We must change our materially based analyses of the world around us*

*to include broader, more multidimensional perspectives.*

*- Albert Einstein*

By reformulating the questions, we aim:

1. To demonstrate how pragmatism, outcomes and implementation cannot be measured by numerical value alone and must make room for a **wider range of measuring values** that are important to implementation and cannot be reduced (e.g., experience, motivation, attitude).
2. To make the process of defining pragmatism more **engaging, interpretive, intuitive, and open** to a wider range of stakeholders.

# Method

| Step 1: We will recruit stakeholders like you from the PPI group and **sent you a questionnaire worksheet** (based on the original questions used to compile the psychometric pragmatic measures scale). We would like you to **fill out the questionnaire** and bring it to the working group.  *Note: The worksheet will ask you to discuss the acceptability of some statements used to assess pragmatism and write comments as to whether you agree or disagree with them and why not. You do not have to answer all of the questions and there are no right or wrong answers. Any thoughts and opinions you may have on the statement will be much appreciated.*  Step 2: Once you’ve had time to go through the worksheet, we will set up **a working group** and invite you to join to discuss the questions and explore the topic more thoroughly. |  |  |
| --- | --- | --- |

# Outcomes

The exercise is designed to highlight the assumptions implicit when using psychometrics and demonstrate some of the complexity that psychometric measures may miss. We wish to get your opinions so that we can assess the inclusivity of Implementation science methodologies and assess if the ways in which we measure implementation outcomes may lead research to be exclusionary.

**Specifically, by engaging the public we wish to consider how to:**

- Make Implementation science **more accessible and easier to comprehend**
- Make complicated topics **more engaged with public interests**
- Make Implementation science **more relevant to everyday experiences and situations**
- Put Implementation science **in the context of wider forces** (rather than the other way around)
- Encourage **more discussion around the subject** (rather than teaching compliance alone)
- Improve **integration between implementation and other disciplines** that have more awareness in the public.

Thank you for your contribution
