# Supplementary material for: Expanding the pragmatic lens in implementation science: why stakeholder perspectives matter
Source: Implement Sci Commun. 2025 Apr 23;6:48. doi: 10.1186/s43058-025-00730-z (PMC12016074; doi:10.1186/s43058-025-00730-z)
Supplement: Supplementary file 2 — Supplementary Material 2. [file 43058_2025_730_MOESM2_ESM.docx]

Work sheet

This exercise sheet has been compiled to engage you in a discussion around Implementation science. We specifically want to hear your opinion on how we measure whether implementation has been successful, and how to make effective methods more usable.

Below are some statements we would like you to consider and offer your opinion on. The questions are based upon current methods to measure usability in Implementation science but have been altered to allow a more diverse range of people to participate.

The questions have been designed to be as easy and relevant as possible but may seem abstract as they have a lot of scientific rationale behind them. Part of this project will assess how translatable this scientific rationale is to people with no prior knowledge or expertise in Implementation science. So, if you do not understand a question, then do not worry about skipping it or missing it out as this will still allow us to understand the relevance of our questions. Please write as much or as little as you like.

Bring what you’ve written to the working group and we will conduct a discussion with you and other participants about the points raised. We aim to create a working group that can explore the issues raised together.

Discussion about **Usefulness**

1. Complicated outcomes that often have social and ethical implications should be ranked numerically.
2. Implementation outcomes should be deemed useful if they justify clinical decision-making.

Discussion about **Compatibility**

1. Implementation outcomes should be measured by how compatible they are with organisational activities.

Discussion about **Acceptability**

1. If individuals feel that an outcome measure biases them to over-report good results over bad results this should not be seen as grounds to discard a measurement on its own.
2. The relative advantage of one measure over another should be rated numerically rather than determined by the specific context of the different scenarios it will be implemented into.
3. Staff and service users’ opinions should be accounted as one part of a numerical rating exercise rather than being central to the whole rating process.
4. The low cost of an implementation outcome should always be accounted when justifying its superiority.

Discussion about **Ease of Use**

1. Outcome measures should be assessed on the accessibility of their language.
2. Low assessor burden (i.e. its ‘easiness’ measured by training, scoring, administration time), is as important as the accuracy when measuring implementation outcomes.
3. The word count of methods used to measure implementation outcomes should be as short as possible, and shorter word counts should be considered more useful than longer ones.
4. When measuring implementation outcomes, if a measure is not completed with ease, it should be considered less useful than the easier ones.
5. When measuring implementation outcomes, a measure should be brief to be deemed easy.
