# Supplementary material for: Expanding the pragmatic lens in implementation science: why stakeholder perspectives matter
Source: Implement Sci Commun. 2025 Apr 23;6:48. doi: 10.1186/s43058-025-00730-z (PMC12016074; doi:10.1186/s43058-025-00730-z)
Supplement: Supplementary file 3 — Supplementary Material 3. [file 43058_2025_730_MOESM3_ESM.docx]

CALL FOR PARTICIPANTS

**The Implementation Science Research theme is looking for members of the public to take part in an exercise about Implementation Science.**

Researchers at the ARC South London are setting up an involvement group to bring a more diverse range of voices into the way we evaluate and measure outcomes in Implementation Science.

Member of the involvement group will work with researchers to:

- Discuss their opinions and attitudes on current methods for measuring implementation success
- Discuss how to make methods more usable

We will make a working group of about 8 people. **No specific expertise is required.**

Members will need to complete a questionnaire and take part in 2 to 3 online group discussions lasting about 1 hour (you will not have to attend any face-to-face meetings).

We will provide £XX of shopping vouchers for every hour you take part.

If you are interested in joining the group please email:
