# Supplementary material for: Expanding the pragmatic lens in implementation science: why stakeholder perspectives matter
Source: Implement Sci Commun. 2025 Apr 23;6:48. doi: 10.1186/s43058-025-00730-z (PMC12016074; doi:10.1186/s43058-025-00730-z)
Supplement: Supplementary file 4 — Supplementary Material 4. [file 43058_2025_730_MOESM4_ESM.docx]

Dear volunteers,

We are enormously grateful to you for offering to get involved in the Study.

We have received a large response and have a limited number of spaces, so we need to perform a selection, and put others on a waiting list.

In the selection process we are guided by two goals:

1) to ensure **representation of diverse communities** and capturing of varied perspectives;

2) to ensure an **in-depth exploration of the topic**, enriched by the contribution of people with the most relevant experience.

The questions below are aligned with these goals:

What is your **gender**? _______________________________________________________

What is your **marital status**?__________________________________________________

What is your **location** (e.g., region)?______________________________________________

What is your **ethnicity**?______________________________________________________

Do you have a **disability** (you do not need to specify which)? ________________________

What is your **age** **range** (e.g., 21-30, 31-40, 41-50, 51-60, 61-70; 71-80; etc.)?___________

What is your **education qualification**?__________________________________________

Are you (please underline):

- A current or previous service-user (e.g., a patient)
- A current or previous carer
- Close to someone who is a service user or a carer (e.g., a family member or a friend)
- Healthcare/social care worker
- Other (please say) ____________________________________________________

Please describe **your experience with the healthcare system**: _____________________________________________________________________________________________________________________________________________________________________________________________________________________________________________________________________________________________________________________________________________________________________________

What is **your experience providing your opinion in the healthcare context**, if any? This can be questionnaires, surveys, interviews, group discussions, where you were asked about the treatment you have undergone, a hospital/clinic where you were treated, healthcare staff behaviours towards you, or other healthcare system aspects. Please provide a brief description:

_____________________________________________________________________________________________________________________________________________________________________________________________________________________________________________________________________________________________________________________________________________________________________________

Which of these dates are convenient for you to meet at 4 pm? (Please underline).

8^th^ of August

10^th^ of August

12^th^ of August

**By filling out this form you are consenting to your data being used for the purposes of participant selection. We guarantee confidential and anonymous treatment of the data you provide and deletion of the data within a couple of weeks of participant selection.**

**Please tick**
