# Supplementary material for: Expanding the pragmatic lens in implementation science: why stakeholder perspectives matter
Source: Implement Sci Commun. 2025 Apr 23;6:48. doi: 10.1186/s43058-025-00730-z (PMC12016074; doi:10.1186/s43058-025-00730-z)
Supplement: Supplementary file 5 — Supplementary Material 5. [file 43058_2025_730_MOESM5_ESM.docx]

Findings

This document has been put together so that you can check the findings of our workshop discussions. Below you will find some text boxes and the findings section. We would like you to read over the findings and then fill in the textboxes about your opinions of the findings.

| Accuracy – does the text accurately express what was discussed/meant during discussions? |
| --- |
| Understandability – can you understand how the findings have been gleamed from the discussions? |
| Agreeability – do you agree with the findings? |
| Importance – what do you think are the most important points in the findings? |
| Additions – is there anything you’d like to add to any of the findings? (including any thoughts you’ve had since then). |
